# A case repot of Merkel cell carcinoma on chronic lymphocytic leukemia: differential diagnosis of coexisting lymphadenopathy and indications for early aggressive treatment

**DOI:** 10.1186/1471-2407-5-106

**Published:** 2005-08-19

**Authors:** KI Papageorgiou, MG Kaniorou-Larai

**Affiliations:** 1 St Andrews Center of Burns and Plastic Surgery, Broomfield Hospital, Court Road, Chelmsford, CM1 7ET, UK; 2 Plastic and Reconstructive Surgery Department, "G. Gennimatas" 6^th ^IKA Oncological Hospital, 11473 Athens, Greece

## Abstract

**Background:**

Chronic lymphocytic leukemia (CLL) is a monoclonal disorder, characterized by a progressive proliferation of functionally incompetent B lymphocytes. There is increased evidence of association between CLL and skin cancers, including the uncommon Merkel cell carcinoma (MCC).

**Case presentation:**

A case report of an 84-year old male, who presented with an aggressively recurrent form of MCC on the lower lip, on the background of an 8-year history of untreated CLL. During the recurrences of MCC, coexisting regional lymphadenopathy, posed a problem in the differential diagnosis and treatment of lymph node involvement. Histopathology and immunoistochemistry showed that submandibular lymphadenopathy coexisting with the second recurrence of MCC, was due to B-cell small lymphocytic lymphoma. The subsequent and more aggressive recurrence of the skin tumor had involved the superficial and deep cervical lymph nodes. Surgical excision followed by involved field radiation therapy has been proven effective for both malignancies.

**Conclusion:**

MCC has a high incidence of regional lymphadenopathy at presentation (12–45%) and even when it arises on the background of chronic leucemia, lymphadenopathy at presentation should be managed agressively with elective lymph node dissection. We overview the postulated correlation between Merkel tumor and CCL, the differential diagnosis of regional lymphadenopathy during the recurrences of the skin tumor and the strategies of treatment

## Background

MCC, is an uncommon tumor, which mostly occurs as an asymptomatic, solitary, firm and red-pink nodule. It has been linked to increased sun exposure [1], both in its anatomic and geographical distribution. Usually is nonulcerated and ranges from 0.8 to 4 cm in diameter [2,3].

Predominantly involves the head and neck region (65%) [4,5], followed by the upper extremities (18%), and the lower extremities (13%) [3]. The precice origin is still controversial. However, the Merkel cell (assumed to be a touch receptor) and the melanocyte are the cutaneous counterparts of the Amine Precurcor Uptake and Decarboxylation (APUD) cells, which are of neuroectodermal origin [6,7].

The tumor is composed of small blue cells with hyperchromatic nuclei and minimal cytoplasm. Mitoses, nuclear fragments and lymphovascular invasion are almost invariable features [8]. Immunoistochemistry for cytokeratin species more typical of simple epitelial cells than keratinocytes, permits a differential identification of Merkel cells in tissue sections [9]. The immunoistochemical profile is characterized of positivity for Neuron-Specific Enolase (NSE), Neurofilament Protein (NFP) and CD57- CD99 [10]. A single punctate zone of cytoplasmic immunoreactivity for cytokeratins especially CK20 is more characteristic [11]. The reaction for CK20 has been used as a finding against metastatic small cell carcinoma of the lung (Cytokeratin 7+ and Thyroid Transcription Factor 1+), small cell melanoma (S100+) and lymphoma (Leucocyte Common Antigen +) [12].

MCC appears to metastasize principally via the lymphatics in a predictable stepwise fashion, with an initial involvement of the regional lymph nodes and subsequent systemic spread [13]. If the lymph nodes are not palpable, the pathological examination of the primary tumor (larger than 2 cm, high mitotic rate and lymphatic invasion) serves as a parameter for the need of lymph node biopsies [5]. Surgical excision with tumor free margins is the primary therapy for stage I-localized disease, with a 64% of survival in 5 years. However recent studies have shown that there is no clear evidence of a difference in survival [2,14], when the margins of resection are less than the 2–3 cm as generally recommended [2,13]

In fact, Mohs micrographic surgery may be more efficacious than wide excision as it inspects all major borders, including the deep margin (MCC often shows extensive vertical growth) and also allows maximal sparing of normal additional tissue, especially in such cosmetically sensitive anatomic areas as the face [15]. A useful adjunct in the treatment of MCC is the sentinel node mapping, which identifies the status of the first draining lymph node and allows to avoid unnecessary lymphadenectomies and the resulting postoperative morbidity [16].

MCC cells are radiosensitive, and several studies have argued for the benefits of radiation therapy not only after resection for local recurrence and palliation but as well as adjuvant treatment after initial surgery with curative intent [12]. Chemotherapy is the least studied theraupetic component and propably is mandatory in unresectable or unmanagable by radiotherapy tumors, as well as in metastatic disease [8,17].

CLL occurs primarily in middle-aged and elderly individuals, with increasing frequency in successive decades of life. It involves slow proliferation and accumulation of incompetent B-lymphocytes and concurrent abnormalities in both humoral and cellular immunity. The majority of patients live 5–10 years, with an initial course that is relatively benign but followed by a terminal, progressive and resistant fase lasting 1–2 years. During the later phase, morbidity is considerable both from the disease and the associated incidence of malignant neoplasms (especially skin cancers as squamous cell carcinoma, Kaposi sarcoma and melanoma) [19]. CLL shares a clinical and morphological overlap with Small Lymphocytic Lymphoma (SLL). In fact if a patient has an absolute lymphocytosis of > 5000/mm^3 ^in the peripheral blood, CLL is diagnosed, regardless of the findings in the lymph node [19].

Treatment of CCL ranges from periodic observation with treatment of infectious, hemorrhagic or immunologic complications to a variety of therapeutic options, including steroids, alkylating agents, purine analogues, combination chemotherapy, monoclonal antibodies and transplant options [20]. As it occurs in an elderly population, progresses slowly and generally is not curable, it is usually treated in a conservative fashion [21]. Involved-field radiation therapy with relatively low doses of radiation can effect an excellent response for both CLL and Small Lymphocytic Lymphoma, especially when the lymphoma cells are contained in one or two areas of lymph nodes in the same part of the body.

## Case presentation

An 84-year old white male, presented at the Department of Dermatology (6^th ^IKA Oncological Hospital of Athens) with a pale, ulcerated lesion (1.3 cm in diameter), on the middle of his lower lip. There was no associated lymphadenopathy and an excisional biopsy was performed. Histopathological and immunoistochemical features revealed a Merkel cell carcinoma (MCC) but as the excision was incomplete the patient was sheduled for a wider excision in the following 2 months. In the meantime, the lesion recurred and the patient returned with a protruding white lesion of 1.1 cm in diameter. There was no associated lynphadenopathy and a wider excision, with an 8 mm margin, was performed. Histopathology confirmed the nature of MCC and the second excision was within healthy margins.

**Figure 1:**
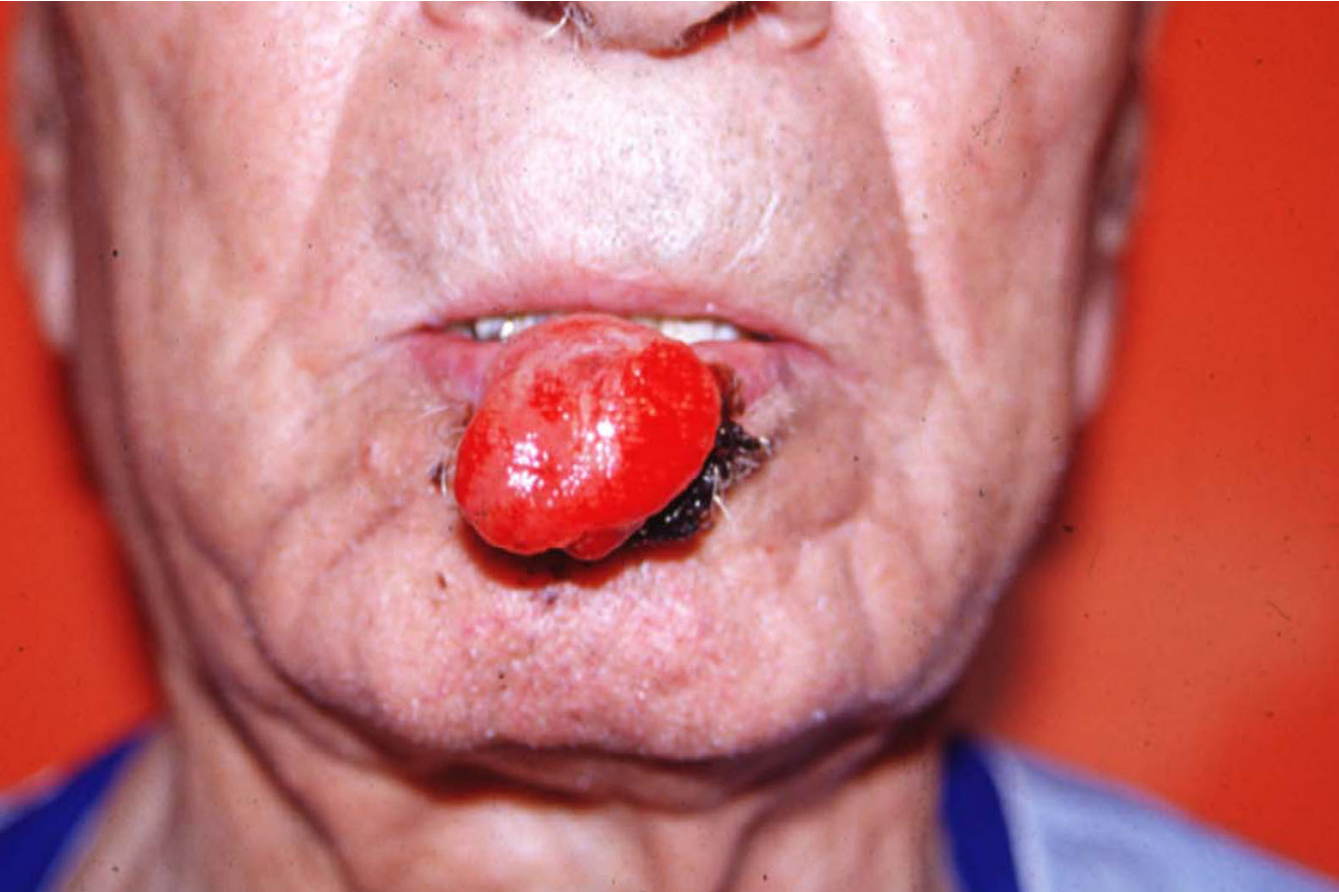
Clinical appearance of second recurrence of Merkel tumor on lower lip.

Two months later, the patient was referred to the Depatment of Plastic Surgery, for another protruding ulcerated lesion, 3 cm in diameter, on his lower lip (Fig 1). On examination, multiple palpable lymph nodes in the submandibular and cervical area (superficial and deep cervical lymphadenopathy) were present.

Past medical history revealed that 8 years earlier, the patient had been diagnosed as having chronic lymphocytic leukemia (CLL), (nodular and intermediate type), but he didn't receive any treatment. CT scan of the head and neck area, showed a soft tissue lobular mass, 3 cm in diameter, on the lower lip, with a possible extension to the mandible. The past medical history of CLL with the recent occurence of MCC posed a problem in the differential diagnosis of the patient's lymphadenopathy. A W-excision (4 × 3.5 × 1 cm) of the lip lesion was performed, with an open biopsy of one submandibular lymph node.

**Figure 2:**
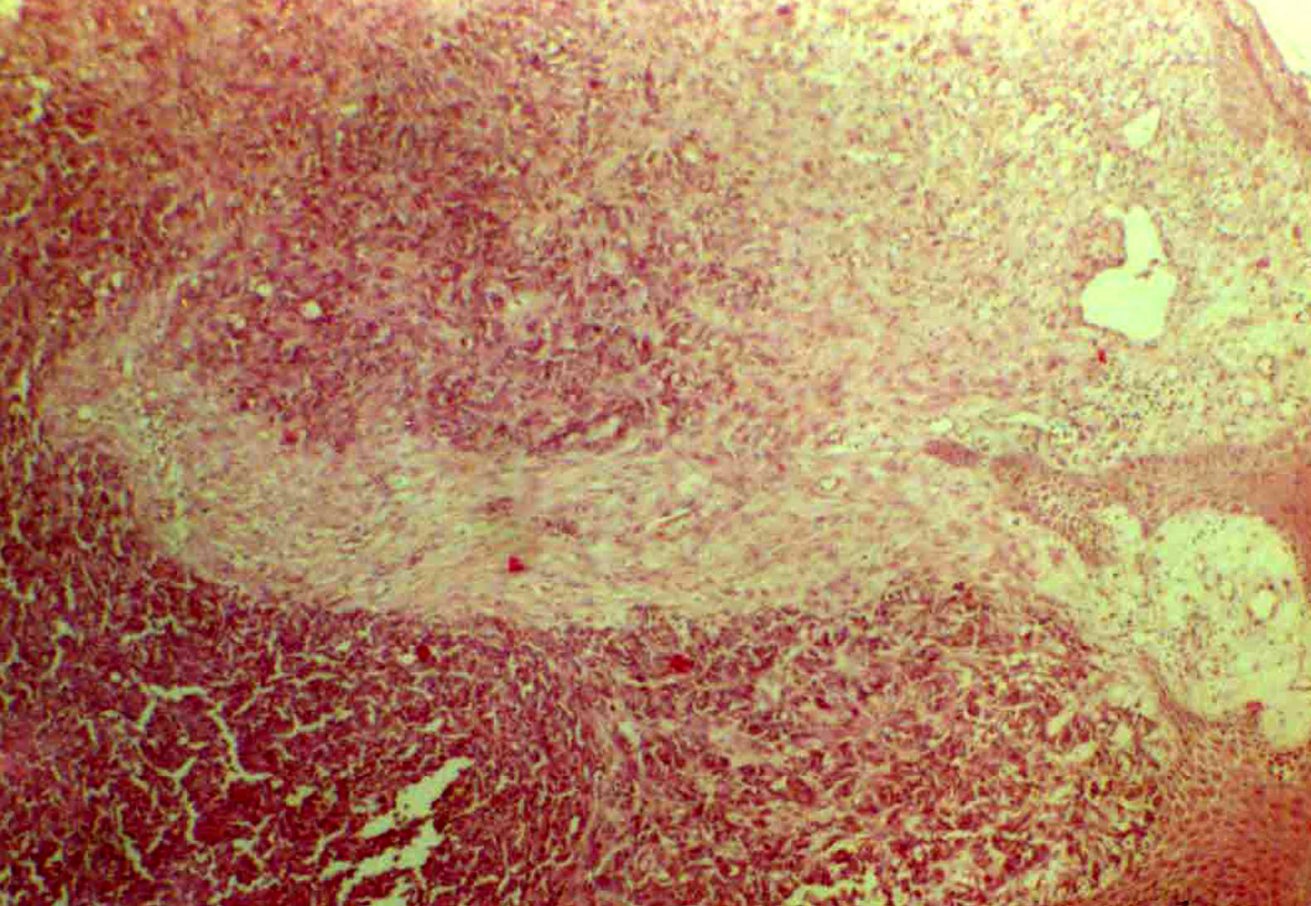
Histopathology showing undifferentiated small cell carcinoma with paucicellular stroma and cells forming nodules or rosettes (haematoxylin and eosin; × 100).

**Figure 3:**
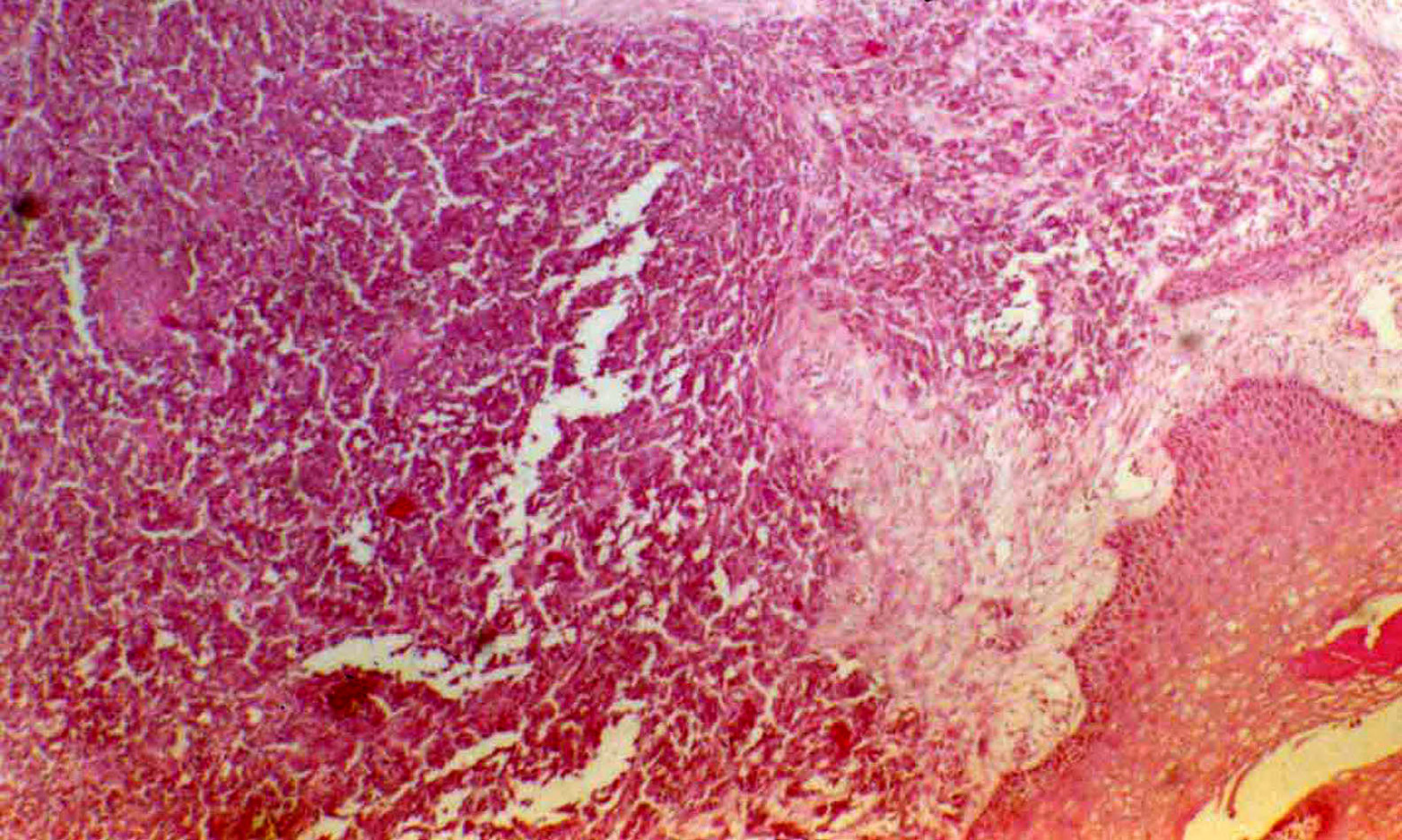
Histopathology showing Merkel cells arranged in rosettes with mitoses present (haematoxylin eosin; × 100).

Histopathology, confirmed recurrence of MCC. An undifferentiated small cell carcinoma with hyaluronated stroma was identified. The cells arranged in nodules or rosettes, had dense nuclear chromatin, with mitoses and nuclear debris which are regular features of MCC (Fig. 2, 3).

Immunoistochemical procedures showed Neuron Specific Enolase (NSE) positivity, while antibodies for Epithelial Membrane Antigen (EMA) and Chromogranin were negative. The excision was described as complete. The submandibular lymph node was positive for malignacy but was associated with the CLL (non Hodgkin's, B cell small lymhocytic lymphoma). There was no evidence of metastatic infiltration by MCC and this was confirmed immunoistochemically with the positive expression of CD5 and CD20 antibody and negative expression of CD10 antibody and NSE. Due to the age of the patient, chemotherapy was not considered.

A month later, a new CT scan of the head and neck, depicted a soft tissue mass, consistent with recurrence of MCC, between the left angle of the mandible and the hyoid bone. Multiple enlarged superficial and deep cervical lymph nodes were present. Fine needle aspiration (FNA) of the submandibular swelling was performed and confirmed MCC (small atypical cells were found, isolated or forming rosettes and exhibited dense core granules of chromatin and scanty cytoplasm). The patient underwent one month of neck radiotherapy with Cobalt 60, a total dose of 4600cGy in 23 days. A further boost of 600cGy in 2 days, on the left submandibular area was administered.

The treatment was successfully completed with full remission of the cervical lymphadenopathy. Two months following the radiotherapy, a new CT scan of the head and neck showed reduction and obscurrence of the pre-existing mass on the left mandibular area, while the lymph nodes were smaller too. The patient is on regular follow up and CLL status is stable with no evidence of progression or further recurrence of MCC 9 months post- radiotherapy.

## Conclusion

Our patient represents another case of MCC arising on CLL and this ocurrence re-inforces the postulated correlation between these 2 malignancies. There are two main aetiological factors associated with increased risk of skin cancers: Ultraviolet radiation and immunosupression. CLL is thought to cause immunosupression and alcylating agents and other immunosuppressant therapies may be involved [22]. A higher incidence of MCC is seen in patients with organ transplantation, with human immunodeficiency virus-1 infection or with advanced cancer and anergic status [23].

On the other side, CLL is relatively common and the association with skin cancers, including the rarer MCC, may be coincidental [24]. The submandibular lymphadenopathy on the second recurrence was unexpectedly due to lymphoma and this complicated the decision on the modality of treatment and further management of the patient. Fortunately both tumors responded to field radiation therapy and on the last follow up there was no evidence of deterioration of the leukemic status or MCC metastatic spread. Probably, a wider excision during the initial diagnosis of the skin tumor (with at least a 1.5 cm margin) [13], should have led to an earlier regression of MCC. However, Gillenwater et al [25] demonstrated no difference in outcome based on margins < 1 cm, 1 to 2 cm and > 2 cm. On the other sider, considering the agressiveness of MCC, radiation therapy should have been involved during the second recurrence, although lymphadenopathy was then positive only for CLL related lymphoma. However, as the subsequent lymph node recurrence of MCC occurred only one month later, it raises the possibility that it was already present when the biopsy was performed and the biopsy result was falsely reassuring.

Complete spontaneous regression constitutes 1.67% of the approximately 600 reported cases of MCC[26]. Therefore, MCC once recognized has to be treated agressively. Studies have shown that the best outcome is obtained in patients with regional disease following lymph node dissection with or without subsequent radiation. Our case report highlights the importance of aggressive treatment of MCC with elective lymph node dissection at presentation, even if the coexisting lymphadenopathy could be related to the coexisting CLL

Certainly, on the background of CLL, clinicopathological features and treatment modalities are more complicated and effectiveness depends on the activity status of the leukemia, which could facilitate recurrence and regional or distant metastatic spread.

## Abreviations

Small Lymphocytic Lymphoma (SLL).

Merkel Cell Carcinoma (MCC)

Chronic Lymphocytic Leucemia (CLL)

## Competing interests

The author(s) declare that they have no competing interests.

## Authors' contributions

KP conceived of the case, participated in the sequence alignment, drafted and revised the manuscript before and following the peer review. M K-l conceived of the case, had access to the data, participated in the sequence alignment and drafted the manuscript.

## Pre-publication history

The pre-publication history for this paper can be accessed here:
